# Altered Neural Basis of the Reality Processing and Its Relation to Cognitive Insight in Schizophrenia

**DOI:** 10.1371/journal.pone.0120478

**Published:** 2015-03-20

**Authors:** Jung Suk Lee, Ji Won Chun, Sang-Hoon Lee, Eosu Kim, Seung-Koo Lee, Jae-Jin Kim

**Affiliations:** 1 Institute of Behavioral Science in Medicine, Yonsei University College of Medicine, Seoul, Rep. of Korea; 2 Department of Psychiatry, Bundang Jesaeng Hospital, Seongnam, Rep. of Korea; 3 Department of Psychiatry, Yonsei University College of Medicine, Seoul, Rep. of Korea; 4 Department of Radiology, Yonsei University College of Medicine, Seoul, Rep. of Korea; West China Hospital of Sichuan University, CHINA

## Abstract

It has been reported that reality evaluation and recognition are impaired in patients with schizophrenia and these impairments are related to the severity of psychotic symptoms. The current study aimed to investigate the neural basis of impairments in reality evaluation and recognition and their relationships with cognitive insight in schizophrenia. During functional magnetic resonance imaging, 20 patients with schizophrenia and 20 healthy controls performed a set of reality evaluation and recognition tasks, in which subjects judged whether scenes in a series of drawings were real or unreal and whether they were familiar or novel. During reality evaluation, patients showed decreased activity in various regions including the inferior parietal lobule, retrosplenial cortex and parahippocampal gyrus, compared with controls. Particularly, parahippocampal gyrus activity was correlated with the severity of positive symptoms in patients. During recognition, patients also exhibited decreased activity in various regions, including the dorsolateral prefrontal cortex, inferior parietal lobule and posterior cingulate cortex. Particularly, inferior parietal lobule activity and posterior cingulate cortex activity were correlated with cognitive insight in patients. These findings provide evidence that neural impairments in reality evaluation and recognition are related to psychotic symptoms. Anomalous appraisal of context by dysfunctions in the context network may contribute to impairments in the reality processing in schizophrenia, and abnormal declarative memory processes may be involved in cognitive insight in patients with schizophrenia.

## Introduction

Reality evaluation is defined as the process of discriminating between things existing outside of oneself and figments of others’ imagination [1]. Our previous study demonstrated that patients with schizophrenia showed impairment in reality evaluation, and this impairment was related to positive symptoms such as hallucinations and delusions [1]. Since hallucinations and delusions may stem from a reality evaluation deficit, determining the neural correlates of the reality evaluation process is important for investigating the pathophysiology of schizophrenia.

During the reality evaluation process, people typically perceive complex features of objects and backgrounds, examine their contexts, store the contexts in short-term memory and compare the contexts with social norms of reality in memories. Thus, the processing of contextual information and relational reasoning, or the ability to consider relationships between multiple mental representations [2], is needed by subjects during the reality evaluation process. More specifically, the context network underlying the appraisal and processing of context mainly includes the medial temporal lobe, retrosplenial cortex and posterior parietal cortex [3–4]. In schizophrenia, decreased hippocampal activity during the processing of contextual information has been reported [5]. In addition, relational reasoning is supported by a network of frontoparietal regions including the frontopolar cortex, dorsolateral prefrontal cortex, inferior frontal gyrus, and inferior parietal lobule [6–8]. A previous neuroimaging study showed abnormal reasoning-related activities in these brain regions in patients with schizophrenia [9].

The reality evaluation process can be related to hallucinations and delusions via abnormal memory processes of real/unreal stimuli. For example, a liberal response bias, a tendency to report imaginary events as real, in a recognition memory task has been reported to be associated with hallucinations [10] and delusions [11]. Because a liberal response bias may stem from a general deficit in the processing of contextual information [12] which may be a prerequisite for reality evaluation, reality evaluation is possibly related to memory processes in schizophrenia. Deficits in memory processes were also suggested to contribute to poor cognitive insight in psychotic patients [13]. Given that the level of cognitive insight was correlated with the severity of psychotic symptoms in schizophrenia [14], anomalous memory processes may underlie the formation of psychotic symptoms. Additionally, a discriminability, accuracy in discriminating targets from distracters, was also found to be related to hallucinations [1]. This finding suggests that low discriminability may make the real stimuli remain less vividly in memories, leading to distortion of reality norms, which then can contribute to the formation or maintenance of hallucinations and delusions. A previous finding that hippocampal hyperactivity during encoding was related to memory deficits and positive symptoms in schizophrenia [15] further supports the association between memory processes and psychotic symptoms.

The aim of this study was to investigate a functional neural basis of impairments in reality evaluation and recognition in schizophrenia using functional magnetic resonance imaging (fMRI). We hypothesized that patients with schizophrenia would exhibit (a) impairment in reality evaluation due to a dysfunction of brain regions related to the processing of context information and relational reasoning; and (b) decreased discriminability and increased liberal response bias due to a dysfunction of memory-related regions during recognition, which would be related to cognitive insight. Additionally, it was hypothesized that altered activities during reality evaluation and recognition in the patients would be interrelated and be associated with the severity of psychotic symptoms.

## Materials and Methods

### Participants and Clinical Measurements

Twenty patients with schizophrenia (10 males) and 20 healthy controls (10 males) participated in this study. All patients were recruited at psychiatric outpatient clinics and were in stable phases of illness. The exclusive diagnoses of schizophrenia in the patient group and the exclusions of psychiatric disorders in the control group were made using the Structural Clinical Interview for DSM-IV [16]. Exclusion criteria included the presence of neurological or significant medical illness and current or past substance abuse or dependence. This study was approved by the institutional review board of Yonsei University Severance Hospital. Written informed consent was obtained from all participants before the study began.

The intelligence scores were assessed by Raven’s progressive matrices [17]. Memory function was assessed using the Rey auditory verbal learning test (RAVLT) [18] and Rey complex figure test (RCFT) [19]. Cognitive insight (i.e., subjects’ self-reflectiveness and their overconfidence in their interpretation of their experiences) was measured using the Beck Cognitive Insight Scale (BCIS) [20], a 15-item self-report questionnaire, consisting of 9 items for self-reflectiveness and 6 items for self-certainty. A composite index of the BCIS for cognitive insight was calculated by subtracting the self-certainty subscale score from the self-reflectiveness subscale score. Patients’ clinical symptoms were rated using the Positive and Negative Syndrome Scale (PANSS) [21].

### Behavioral Task

**Fig 1:**
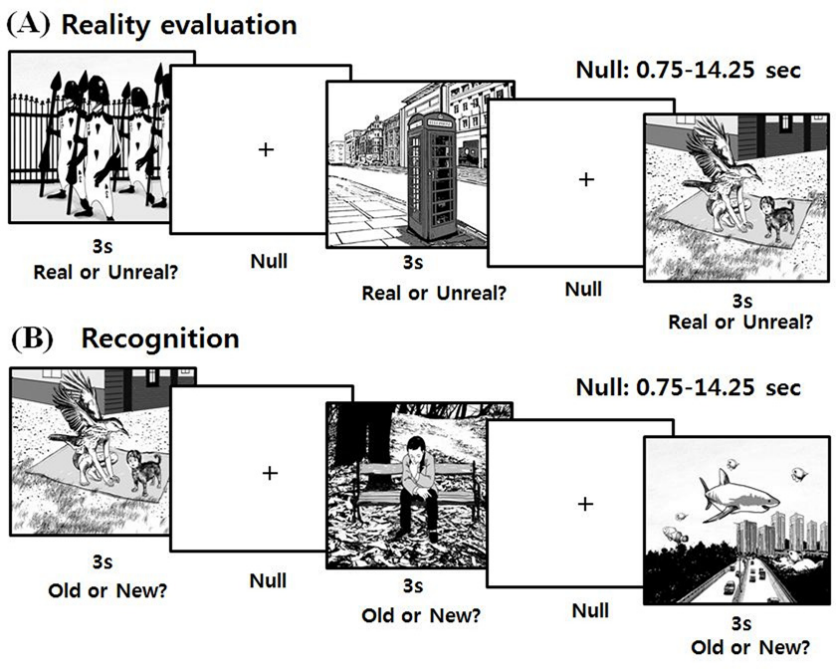
Sequences of the behavioral tasks.

The fMRI task of event-related design was divided into two parts (Fig. 1): (i) a reality evaluation task, and (ii) a recognition task. We used a previously validated set of 36 real and 36 unreal pictures [1] as stimuli. In the reality evaluation task, 18 real and 18 unreal pictures were presented twice serially and randomly to subjects, who were instructed to click the left mouse button if the picture was perceived as real or the right mouse button if the picture was perceived as unreal. To enhance the ecological validity of the procedure, subjects were not informed that recognition would be assessed on the basis of task performance. The recognition task was performed 15 min after the reality evaluation task. While viewing real or unreal pictures, subjects were instructed to click the left mouse button if the picture had been presented during the reality evaluation task (OLD) or the right mouse button if the picture had not been presented (NEW). Thirty six real (18 OLD and 18 NEW) and 36 unreal (18 OLD and 18 NEW) pictures were used in the recognition task. The orders of presentation of the pictures were counterbalanced across subjects. In both tasks, all pictures were presented for 3 sec, and the null events were varied from 0.75 sec to 14.25 sec. After fMRI scanning, subjects were instructed to rate valence of the pictures (positive: 1, neutral: 0, and negative: –1).

### Image Acquisition

MRI data were acquired on a 3 T MR scanner (Intra Achieva; Philips Medical System, Best, Netherlands). Thirty-eight contiguous 3.5-mm-thick axial slices were collected using a single-shot echo planar imaging sequence depicting the blood-oxygenation-level-dependent signal (echo time = 30 ms; repetition time = 3,000 ms; flip angle = 90°; field of view = 220 mm; and image matrix = 128×128). Axial 1.2-mm-thick T1-weighted images (echo time = 4.6 ms; repetition time = 9.703 ms; flip angle = 30°; field of view = 220 mm; and image matrix = 256×256) were also collected.

### Behavioral Data Analysis

Clinical data were compared between groups using two-sample *t*-tests. Behavioral performance of the reality evaluation task was measured by the level of accuracy, whereas that of the recognition task was counted by discriminability and response bias in accordance with the Two-High Threshold Theory [22]. Discriminability reflected accuracy in discriminating target pictures from distracters (rate of correct recognitions of target pictures minus rate of false recognitions of nontarget pictures). Response bias, the tendency to make false recognitions of unpresented pictures, was calculated as FA/(1−(H—FA)): the hit (H) and false alarm rates (FA). Values greater than 0.5 were classified as “liberal” bias (i.e., a tendency to say “yes” when unsure), whereas values below 0.5 were classified as “conservative” bias (i.e., a tendency to say “no” when unsure). These behavioral performances and valence rating were analyzed by repeated measures ANOVA with the group (patients or controls) as the between-subject factor and the condition (real or unreal) as the within subject factor, which was followed by *post hoc* least significant difference (LSD) test. In addition, discriminability and response bias were reanalyzed using RCFT delayed recall score as a covariate. Statistical significance was set at *P* < 0.05.

### Neuroimaging Data Analysis

Preprocessing and analysis of the neuroimaging data were performed using Statistical Parametric Mapping 8 (SPM8). The first four volumes of the functional data were discarded to allow signal equilibration. After correcting for slice acquisition time differences, the functional images were realigned to eliminate head movement effects, co-registered to the T1-weighted image, and spatially normalized using nonlinear transformation functions, which were obtained by registering individual T1-weighted images to a Montreal Neurological Institute (MNI) template. These normalized data were smoothed with an 8 mm full-width-at-half-maximum Gaussian filter.

Individual contrast maps were generated by contrasting the unreal condition with the real condition for each of the reality evaluation and recognition tasks at the first-level analysis and the movement parameters obtained from the realignment procedure were included as regressors to account for any residual effects of head motion. Linear contrasts of subject-specific parameter estimates for conditions of interest were taken to a second-level random-effects model. Second-level analyses were performed by 2 (group) x 2 (condition) full factorial model, in which a covariate was the RCFT copy score for the reality evaluation task and the RCFT delayed recall score for the recognition task. The statistical significance was set at uncorrected *P* < 0.001 with more than 10 contiguous voxels to grasp the trend of group differences, and then 10,000 Monte Carlo simulations were conducted and the critical cluster size was determined at family-wise error-corrected *P* < 0.05 via AlphaSim (http://afni.nimh.nih.gov/afni/doc/manual/AlphaSim) to correct for the problem of multiple comparisons. The percent signal change (PSC) in the clusters showing significant main effect of group or group x condition interaction was calculated using MarsBaR (version 0.42, http://marsbar.sourceforge.net/). The PSC in the clusters for the contrast of unreal *versus* real condition was used for the subsequent correlation analyses. Spearman correlation analyses were performed between the regional PSCs and BCIS scores in patients and controls, separately, and between the regional PSCs and PANSS positive scores in patients. Correlation analyses were also performed between the regional PSCs during reality evaluation and those during recognition in each group.

## Results

### Demographic and Clinical Characteristics

There were no significant differences between the patient and control groups in terms of gender and age (patients, 37.1 ± 6.5 years; controls, 36.7 ± 7.1 years). Patients (13.6 ± 1.6 years) had received significantly less years of education than controls (14.9 ± 1.8 years, *P* = 0.03). The mean ratings of positive, negative, and general symptom subscale scores of the PANSS in patients were 12.4 ± 4.6, 13.0 ± 4.7, and 27.1 ± 7.6, respectively. Their mean duration of illness was 11.6 ± 5.1 years and their mean hospitalization number was 3.2 ± 1.7. All patients were medicated with one or two antipsychotics, and the mean chlorpromazine-equivalent dose for the medications was 399.6 ± 291.9 mg.

### Cognitive and Behavioral Measurements

As shown in Table 1, the raw score of the Raven’s Progressive Matrices did not differ between patients and controls. However, patients showed significantly lower RAVLT recognition scores (*P* < 0.001) and RCFT immediate (*P* < 0.001) and delayed recall scores (*P* < 0.001) than controls. The RCFT copy scores were not different between the two groups. The BCIS self-certainty, self-reflectiveness and composite scores did not show any group difference.

**Table 1:** Clinical and behavioral data of subjects.

	Patients	Controls	*t*/*F*	*P* value
	(n = 20)	(n = 20)		
RPM raw score	47.5±9.3	50.4±7.8	−1.05	0.30
RAVLT recognition	11.7±2.7	14.6±0.6	−4.55	<0.001
RCFT copy	31.3±6.3	33.5±2.0	−1.54	0.13
RCFT immediate recall	16.6±7.4	26.0±5.3	−4.55	<0.001
RCFT delayed recall	15.8±6.7	25.5±5.3	−5.02	<0.001
BCIS self-certainty	5.9±3.0	7.1±2.8	−1.36	0.18
BCIS self-reflectiveness	9.6±4.6	9.2±3.3	0.32	0.75
BCIS composite	3.7±4.6	2.1±2.8	1.38	0.18
Reality evaluation accuracy			5.53	0.02
Real condition	0.83±0.17	0.91±0.08		
Unreal condition	0.86±0.13	0.93±0.08		
Discriminability			11.69	0.002
Real condition	0.65±0.25	0.84±0.10		
Unreal condition	0.69±0.23	0.88±0.08		
Response bias			39.74	<0.001
Real condition	0.67±0.27	0.88±0.26		
Unreal condition	0.41±0.26	0.32±0.34		
Valence			290.16	<0.001
Real condition	0.73±0.39	0.86±0.16		
Unreal condition	−0.58±0.30	−0.78±0.31		

RPM, Raven’s Progressive Matrices; RAVLT, Rey Auditory Verbal Learning Test; RCFT, Rey Complex Figure Test; BCIS, Beck Cognitive Insight Scale.

For reality evaluation accuracy, there was a significant main effect of *group* [*F*(1,37) = 5.53, *P* = 0.02], but no significant main effect of *condition* or interaction effect of *group x condition*. Accuracy was significantly lower in patients than in controls (*P* = 0.02). Regarding discriminability, there was a significant main effect of *group* [*F*(1,37) = 11.69, *P* = 0.002], but no significant main effect of *condition* or interaction effect of *group x condition*. Discriminability was significantly lower in patients than in controls (*P* = 0.002). After controlling for the RCFT delayed recall score, these results for discriminability were not changed with only a significant main effect of *group* [*F*(1,35) = 9.14, *P* = 0.005]. For response bias, there was a significant main effect of *condition* [*F*(1,37) = 39.74, *P* < 0.001] and there was a significant interaction effect of *group x condition* [*F*(1,37) = 5.35, *P* = 0.03], but no significant main effect of *group*. Response bias was significantly higher for the real condition than for the unreal condition in controls (*P* < 0.001), but not significantly different in patients. After controlling for the RCFT delayed recall score, there were no significant main effect of *group* or *condition* and interaction effect of *group x condition* for response bias. In addition, the demographic variables including years of education and cognitive performances showed no significant correlation in each group.

For valence of the stimulus pictures, there was a significant main effect of *condition* [*F*(1,37) = 290.16, *P* < 0.001], but no significant main effect of *group* or interaction effect of *group x condition*. Valence was significantly lower for the unreal condition than for the real condition (*P* < 0.001).

### Brain Activity during Reality Evaluation

**Fig 2:**
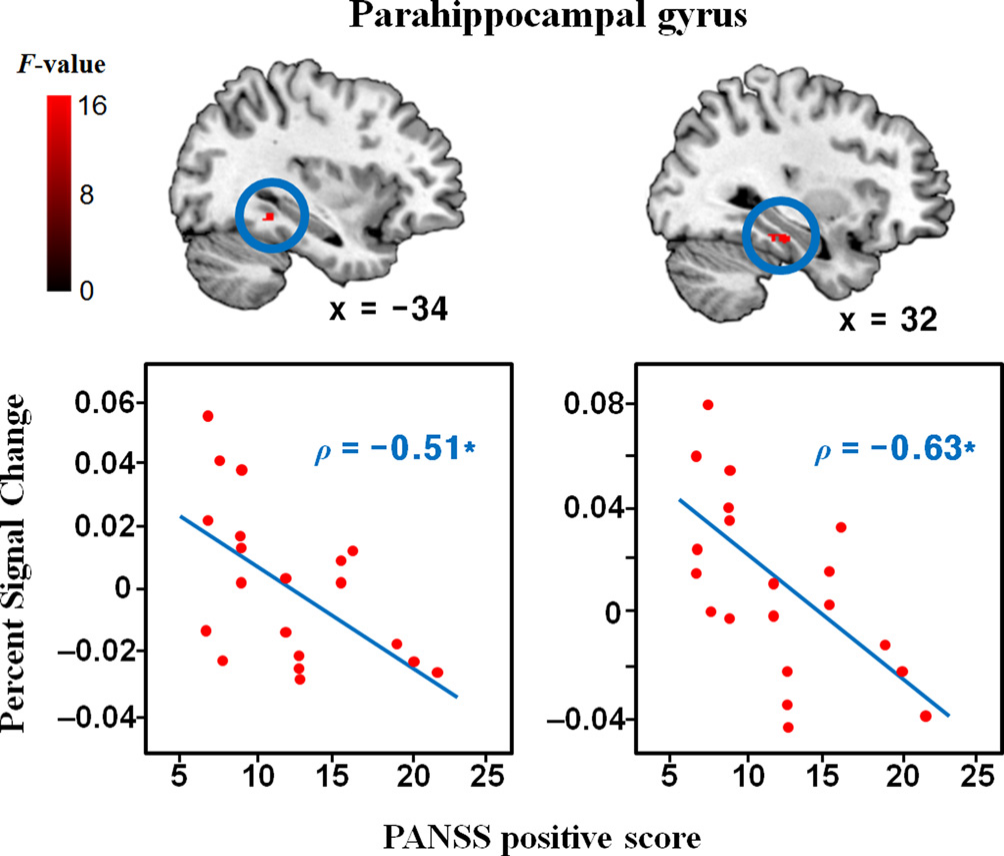
Brain regions showing a significant group difference in reality evaluation-related activity and their correlations with the Positive and Negative Syndrome Scale (PANSS) positive scores. Scatter plots depict the relationship between the regional percent signal change and PANSS positive scores in patients. The percent signal changes of the left and right parahippocampal gyri during reality evaluation were significantly lower in patients than in controls, and they were significantly correlated with the PANSS positive scores in patients *Significant finding at *P* < 0.05.

**Fig 3:**
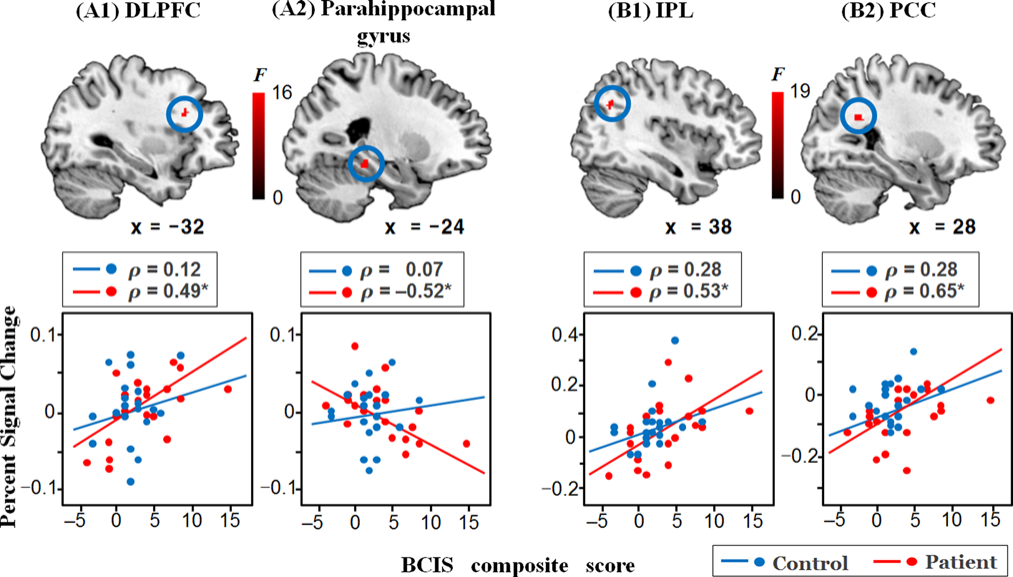
Brain regions showing a significant group difference and correlation with the Beck Cognitive Insight Scale (BCIS) composite scores. Scatter plots depict the relationship between the regional percent signal change and BCIS composite scores in patients and controls. The percent signal changes of the left dorsolateral prefrontal cortex (DLPFC) (A1) and left parahippocampal gyrus (A2) during reality evaluation were significantly lower in patients than in controls, and they were significantly correlated with the BCIS composite scores in patients, but not in controls. The percent signal changes of the right inferior parietal lobule (IPL) (B1) and right posterior cingulate cortex (PCC) (B2) during recognition were significantly lower in patients than in controls, and they were significantly correlated with the BCIS composite scores in patients, but not in controls. *Significant finding at *P* < 0.05.

Brain regions showing a main effect of *group* are listed in Table 2. At the corrected significance level, compared to controls, patients exhibited significantly decreased activity in the right inferior parietal lobule, right retrosplenial cortex, bilateral (x = -34 and x = 32) parahippocampal gyri, and right caudate. Additional regions including the left dorsolateral prefrontal cortex, left (x = -24) parahippocampal cortex, and left putamen were observed at the uncorrected significance level. There was no brain region showing significantly increased activity in patients than in controls. The significant *group x condition* interaction was found only in the right caudate. In the correlations between the regional PSCs and psychotic symptom measurements in patients (Fig. 2), the PANSS positive scores were correlated with only left (x = -34) and right (x = 32) parahippocampal gyrus activity (*ρ* = -0.51, *P* = 0.02; *ρ* = -0.63, *P* = 0.003, respectively). The BCIS composite scores were correlated with left dorsolateral prefrontal cortex activity (*ρ* = 0.49, *P* = 0.03) and left (x = -24) parahippocampal gyrus activity (*ρ* = -0.52, *P* = 0.02) in patients (Fig. 3A1 and 3A2), but not with any regional activity in controls. The BCIS self-reflectiveness scores were correlated with left (x = -24) parahippocampal gyrus activity (*ρ* = -0.72, *P* < 0.001) in patients and left middle occipital gyrus activity (*ρ* = -0.57, *P* = 0.01) in controls.

**Table 2:** Brain regions showing a significant main effect of *group* or *group x condition* interaction during reality evaluation and recognition.

		MNI Coordinates			
Regions (Brodmann area)	*F*	x	y	z	Nvox
*During reality evaluation (controlled for the RCFT copy scores)*					
*Main effect of group*					
Schizophrenia < Control					
Dorsolateral prefrontal cortex (9), left	15.1	-32	20	28	10
Inferior parietal lobule (39), right*	19.9	44	-70	24	56
Retrosplenial cortex (29), right*	20.0	20	-46	14	68
Parahippocampal gyrus (36), left*	15.7	-34	-44	-12	71
Parahippocampal gyrus (36), left	15.9	-24	-36	-10	15
Parahippocampal gyrus (36), right*	16.9	32	-28	-18	44
Middle occipital gyrus (19), left	15.7	-22	-78	20	20
Caudate, right*	24.8	8	24	6	110
Putamen, left	19.1	-28	4	-14	15
Schizophrenia > Control					
None					
*Group x condition interaction*					
Caudate, right*	24.2	8	24	10	41
*During recognition (controlled for the RCFT delayed recall scores)*					
*Main effect of group*					
Schizophrenia < Control					
Dorsolateral prefrontal cortex (9), right*	23.8	44	28	22	62
Inferior parietal lobule (39), right*	17.3	38	-64	32	41
Inferior parietal lobule (40), left	18.3	-46	-44	46	19
Parietal operculum (43), left	17.1	-38	-10	24	15
Posterior cingulate cortex (23), right*	19.0	28	-52	26	26
Parahippocampal gyrus (36), left	20.3	-36	-6	-26	22
Premotor cortex (6), left*	29.4	-24	-6	48	59
Premotor cortex (6), right*	16.1	28	10	46	56
Motor cortex (4), left	17.1	-42	-2	24	14
Motor cortex (4), right*	20.6	42	2	28	47
Somatosensory cortex (2), left	21.5	-52	-12	20	12
Fusiform gyrus (37), left	16.6	-50	-52	-14	18
Middle occipital gyrus (19), right*	24.3	44	-60	14	57
Inferior occipital gyrus (19), right*	27.4	38	-72	-8	75
Schizophrenia > Control					
None					

MNI, Montreal Neurological Institute; Nvox, number of voxels; RCFT, Rey Complex Figure Test

All regions listed had the significance level of uncorrected P < 0.001 and above 10 voxels.

*significant at AlphaSim-corrected *P* < 0.05.

### Brain Activity during Recognition

At the corrected significance level, compared to controls, patients showed significantly decreased activity in the right dorsolateral prefrontal cortex, right inferior parietal lobule, right posterior cingulate cortex, bilateral premotor cortices, right motor cortex, and right middle and inferior occipital gyri (Table 2). Additional regions including the left inferior parietal lobule, left parietal operculum, and left parahippocampal cortex were observed at the uncorrected significance level. There was no brain region showing significantly increased activity in patients than in controls or significant *group x condition* interaction. In the correlation analyses, there was no brain region showing significant correlation with psychotic symptom measurements in patients. The BCIS composite scores were correlated with right posterior cingulate cortex activity (*ρ* = 0.65, *P* = 0.002) and right inferior parietal lobule activity (*ρ* = 0.53, *P* = 0.02) in patients (Fig. 3B1 and 3B2), but not with any regional activity in controls. The BCIS self-reflectiveness scores were correlated with right inferior parietal lobule activity (*ρ* = 0.59, *P* = 0.01) in patients and right motor cortex activity (*ρ* = 0.51, *P* = 0.02) in controls.

### Regional Correlations between Reality Evaluation and Recognition

**Fig 4:**
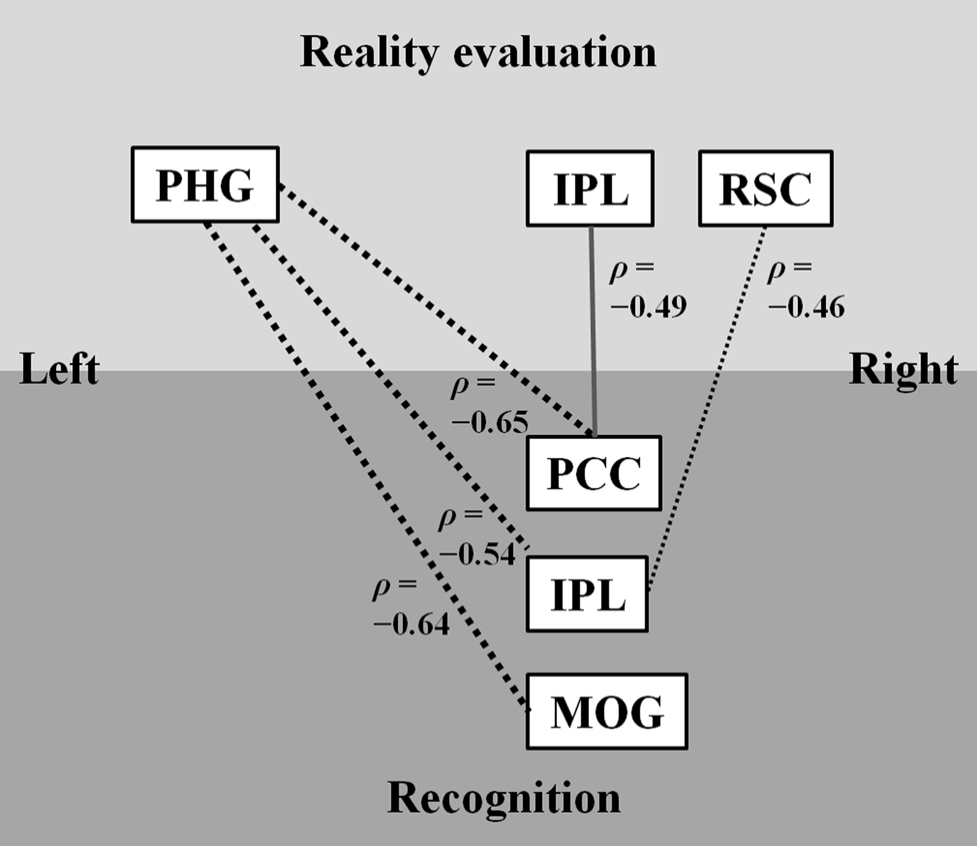
The relationship between reality evaluation-related activity and recognition-related activity. Dotted lines represent significant correlations in patients, whereas a solid line indicates a significant correlation in controls. Particularly, thick dotted line represents a significant difference in correlation coefficients between patients and controls. PHG, parahippocampal gyrus; IPL, inferior parietal lobule; RSC, retrosplenial cortex; PCC, posterior cingulate cortex; MOG, middle occipital gyrus.

As shown in Fig. 4, significant regional correlations in patients were found between left (x = -24) parahippocampal gyrus activity during reality evaluation and activity of the right posterior cingulate cortex (*ρ* = -0.65, *P* = 0.002), right inferior parietal lobule (*ρ* = -0.54, *P* = 0.01), and right middle occipital gyrus (*ρ* = -0.64, *P* = 0.002) during recognition, and between right retrosplenial cortex activity during reality evaluation and right inferior parietal lobule activity (*ρ* = -0.46, *P* = 0.04) during recognition. In controls, there were significant correlations between right inferior parietal lobule activity during reality evaluation and right posterior cingulate cortex activity (*ρ* = -0.49, *P* = 0.03) during recognition. Comparisons of correlation coefficients between the two groups showed a significant difference in the correlations between left (x = -24) parahippocampal gyrus activity during reality evaluation and activity of the right posterior cingulate cortex (*P* = 0.02), right inferior parietal lobule (*P* = 0.03), and right middle occipital gyrus (*P* = 0.03) during recognition.

## Discussion

Consistent with our hypothesis and in agreement with our previous study [1], we found that patients with schizophrenia were less accurate during reality evaluation than healthy controls, suggesting that patients have deficits in reality evaluation. In addition, patients showed significantly decreased discriminability compared to controls, and this group difference remained significant even after controlling for the RCFT delayed recall scores. This finding suggests that recognizing real/unreal stimuli was more challenging to patients than the RCFT recall procedure. Compared to the figure of the RCFT, the real/unreal stimuli would require the processes of understanding the context, and this additional required process might make the recognition process more difficult.

Patients showed decreased activity in the bilateral parahippocampal gyri during reality evaluation. As one of the major components of the context network, the parahippocampal gyrus is involved not only in declarative memory process, but also in context appraisal [3]. As a recent study demonstrated that patients with schizophrenia were impaired in the context processing and this impairment could lead to poor integration of information during encoding [23], deficient context appraisal may prevent a subject from getting sufficient information to judge the reality of stimuli, eventually disturbing the reality evaluation process. Furthermore, parahippocampal gyrus activity during reality evaluation was negatively correlated with the PANSS positive scores, and this is consistent with previous studies which found the significant association between abnormal activation in the parahippocampal gyrus and positive symptoms in schizophrenia [24–25]. Because dysfunctional parahippocampal gyrus has been related to impaired context appraisal [25–26], this dysfunction may lead to errors in validating external sensory inputs and internal mental representation, predisposing patients with schizophrenia to hallucinations and delusions. Taken together, functional abnormalities in the parahippocampal gyrus may play an important role in the formation of psychotic symptoms in schizophrenia.

Decreased activity in the retrosplenial cortex during reality evaluation in patients can also be relevant to impairment in context appraisal in that this region has been regarded as a component of context network [4,27]. The retrosplenial cortex has been suggested to mediate both spatial and non-spatial contextual processing and contribute to contextual elements of episodic memory [28]. Although both the parahippocampal gyrus and retrosplenial cortex are involved in the processing of contextual information, these regions may play a different role. For example, a previous fMRI study proposed that the retrosplenial cortex represents abstracted properties of an object context, whereas the parahippocampal gyrus processes physical properties [29]. Therefore, parahippocampal and retrosplenial dysfunctions may be related to reality evaluation impairment in schizophrenia through separate involvement in context appraisal.

Compared to controls, decreased activity in the inferior parietal lobule during reality evaluation was found in patients. In addition, although not significant after AlphaSim correction, patients showed decreased activity in the dorsolateral prefrontal cortex during reality evaluation. Because the inferior parietal lobule and dorsolateral prefrontal cortex are the brain regions subserving relational reasoning [30], this finding can be interpreted in terms of reasoning abnormality. During reality evaluation process, subjects should judge the reality of stimuli according to norms of reality [1], which is closely related to relational reasoning. Our data suggest that anomalous reasoning may contribute to impairment in reality evaluation in schizophrenia. The BCIS composite scores were correlated with dorsolateral prefrontal activity in patients. Cognitive insight reflects the ability to consider a number of information, perspectives and alternative hypotheses at the same time and process relationships between them to make correct judgments [20]. Because this ability would need the relational reasoning process, our findings suggest that poor cognitive insight may be related to reasoning abnormality in schizophrenia.

During recognition, patients exhibited decreased activity in the inferior parietal lobule and posterior cingulate cortex. Recent neuroimaging studies have demonstrated memory-related activation in these two regions [31]. The inferior parietal lobule is typically involved in episodic memory retrieval [32,33], probably by bottom-up attention processes, which refers to attentional guidance purely by the saliency of incoming information and is mediated by the ventral fronto-parietal system [34]. The posterior cingulate cortex receives input from the posterior hippocampus [35] and also plays a role in the memory process [36]. In addition, our study showed that inferior parietal lobule activity and posterior cingulate cortex activity were positively correlated with the BCIS composite scores in patients. These results further supports a previous assertion that cognitive insight in patients with schizophrenia may rely on declarative memory processes whereby current experiences are appraised based on previous ones [13]. Since poorer cognitive insight was related to more severe psychotic symptoms in schizophrenia [14], disturbances in declarative memory processes might be associated with hallucinations and delusions in patients with schizophrenia, which is in line with our previous finding that discriminability for the real pictures was correlated with the severity of hallucinations in patients [1].

In patients, decreased activity of the left parahippocampal gyrus during reality evaluation was related to increased activity of the right inferior parietal lobule and right posterior cingulate cortex during recognition, while decreased activity of the right retrosplenial cortex during reality evaluation was related to increased activity of the right inferior parietal lobule. Decreased activity of the parahippocampal gyrus and retrosplenial cortex was related to impaired context appraisal, and increased activity in the inferior parietal lobule and posterior cingulate cortex was associated with better cognitive insight. The parahippocampal gyrus has reciprocal connections with the inferior parietal lobule, posterior cingulate cortex and retrosplenial cortex [37]. Thus, these findings suggest that the inferior parietal lobule and posterior cingulate cortex might be activated to enhance cognitive insight and to compensate for impaired processing of context caused by deficient activation of the parahippocampal gyrus and retrosplenial cortex. The negative correlation between left parahippocampal gyrus activity and the BCIS composite score in patients might be due to the relationship between increased parahippocampal gyrus activity and decreased inferior parietal lobule and posterior cingulate cortex activity, which showed a positive correlation with the BCIS composite score.

**Fig 5:**
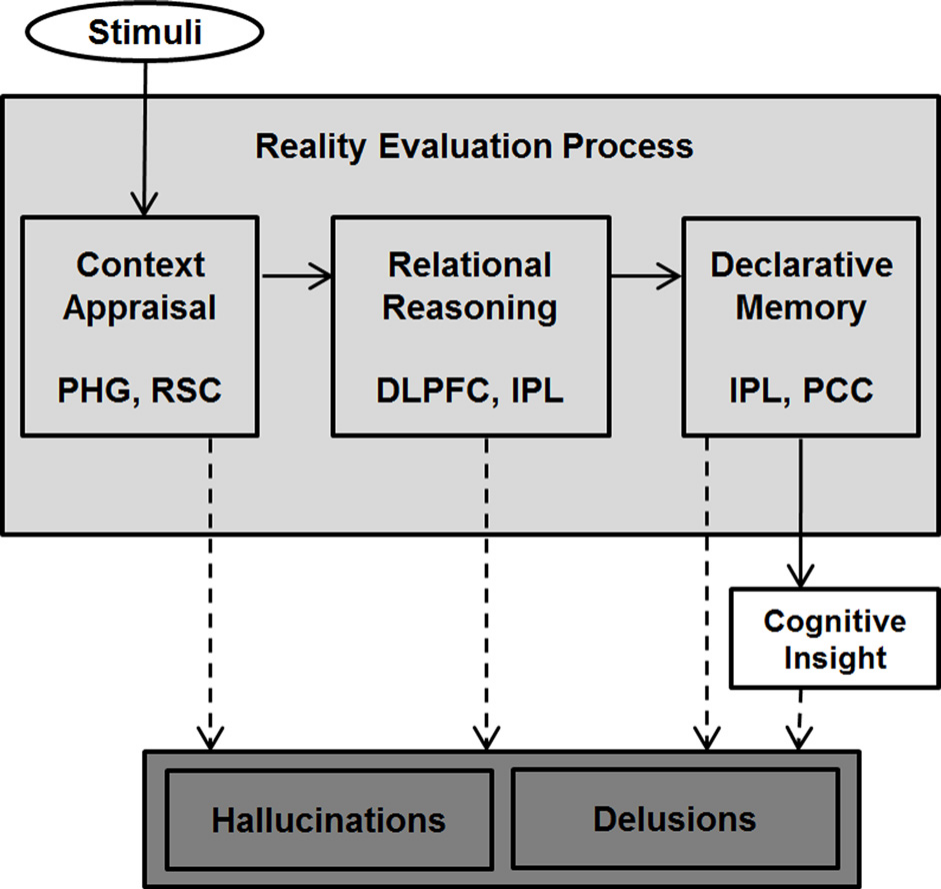
Proposed model of the reality evaluation process. PHG, parahippocampal gyrus; RSC, retrosplenial cortex; DLPFC, dorsolateral prefrontal cortex; PCC, posterior cingulate cortex; IPL, inferior parietal lobule.

Based on previous studies and our results, we propose a model of the reality evaluation process (Fig. 5), which consists of three phased processes contributing to the formation and maintenance of hallucinations and delusions. The first process—“context appraisal”—is thought to be mediated by the context network including the parahippocampal gyrus and retrosplenial cortex. The second process, “relational reasoning,” is involved in judging the reality of stimuli according to norms of reality, which is suggested to be mediated by the dorsolateral prefrontal cortex and inferior parietal lobule. The third process, “declarative memory,” is thought to be mediated by the inferior parietal lobule and posterior cingulate cortex. This process is closely related to cognitive insight, which may be involved in improvement of hallucinations and delusions. Thus, enhancing cognitive insight may be compensation for reality evaluation impairment in schizophrenia.

Several limitations should be acknowledged. First, patients in this study were taking antipsychotic medications and in a state of chronic illness with multiple episodes. Therefore, it is unclear whether the behavioral and neural impairments of reality evaluation and recognition are their trait or state. Although no evidence exists about the relationship between antipsychotics and reality evaluation process, it is possible that antipsychotic medication affects regional brain activity related to the reality processing. Second, viewing a drawing in the MRI scanner can be different from reality monitoring in real life. Third, cognitive measurements were limited to reasoning and memory. Although other cognitive functions such as executive functions and attention were not considered to be a main confounding factor, they can affect task performance.

In conclusion, this study demonstrated that impairments in reality evaluation and recognition appear to be related to abnormal functioning in the context network, and anomalous appraisal and processing of context may contribute to positive symptoms in schizophrenia. The significant association between activity in memory-related brain regions and cognitive insight further suggests that declarative memory processes may play an important role in cognitive insight in patients with schizophrenia. In addition, memory-related brain regions may be activated to enhance cognitive insight and to compensate for reality evaluation impairment. The current study provides new insights into the pathophysiology of positive symptoms in schizophrenia.
